# Angiotensin II infusion in COVID-19-associated vasodilatory shock: a case series

**DOI:** 10.1186/s13054-020-02928-0

**Published:** 2020-05-15

**Authors:** Alberto Zangrillo, Giovanni Landoni, Luigi Beretta, Federica Morselli, Ary Serpa Neto, Rinaldo Bellomo, the COVID-BioB Study Group, Anna Mara Scandroglio, Sergio Colombo, Antonio Dell’Acqua, Paolo Silvani, Evgeny Fominskiy, Giacomo Monti, Maria Luisa Azzolini, Antonio Bellantoni, Cristina Barberio, Gabriele Valsecchi, Omar Saleh, Gaetano Lombardi, Moreno Tresoldi, Paolo Scarpellini, Lorenzo Dagna, Fabio Ciceri, Yanase Fumitaka

**Affiliations:** 1 Department of Anesthesia and Intensive Care, IRCCS San Raffaele Scientific Institute, Via Olgettina 60, 20132 Milano, Italy; 2 Australian and New Zealand Intensive Care Research Centre (ANZIC-RC), School of Public Health and Preventive Medicine, Monash University, Melbourne, Australia; 3 Department of Critical Care Medicine, Hospital Israelita Albert Einstein, Sao Paulo, Brazil; 4 Department of Intensive Care, Austin Hospital, Melbourne, Australia; 5 Department of Intensive Care, Royal Melbourne Hospital, Melbourne, Australia; 6 Centre for Integrated Critical Care, School of Medicine, The University of Melbourne, Melbourne, Australia

Two thirds of ventilated COVID-19 patients require vasopressor support [1]. Recommended vasopressors include norepinephrine and vasopressin. Recently, based on a randomized trial [2], angiotensin II (ANGII) was FDA- and EMA-approved for catecholamine-resistant vasodilatory shock. ANGII use as primary vasopressor for vasodilatory shock has never been reported, let alone for COVID-19-associated vasodilatory shock. ANGII may be logical in this setting. It specifically assists patients recently exposed to angiotensin-converting enzyme inhibitors [2, 3] and increases the internalization and downregulation of angiotensin-converting enzyme 2 [4], the receptor for COVID-19. Its use may also inform the debate about the risks and benefits of angiotensin receptor blockers in COVID-19-infected patients [5]. In this pilot compassionate-use case series, we used ANGII either as primary or rescue vasopressor in ventilated patients with COVID-19-associated vasodilatory shock and assessed the course of key physiological variables during the first 48 h of treatment.

We studied a cohort of consecutive ventilated patients in COVID-19-dedicated ICUs at San Raffaele Scientific Institute, Milan, Italy. Patients had vasodilatory shock and COVID-19-related infection (positive viral RNA biospecimen and typical clinical and radiological features). The Ethics Committee approved compassionate use of the drug.

All cases received commercial ANGII (Giapreza®, La Jolla San Diego, CA) as continuous infusion started at 20 ng/kg/min and titrated to a MAP target > 65 mmHg. We collected key data before and during 48 h of angiotensin II infusion.

Over 6 days (March 12 to March 18, 2020) we treated 16 patients, 10 with ANGII as first-line agent, five as second-line agent (Table 1), and one patient with unobtainable data. ANGII dose was relatively constant. MAP and urine output remained stable; lactate and creatinine increased and C-reactive protein decreased (Table 1). However, the SpO_2_/FiO_2_ ratio increased significantly with a decrease in FiO_2_ and PEEP (Fig. 1). At latest follow-up (1 week), 14 patients were alive.

**Table 1:** Baseline characteristics and physiological changes in treated patients

	Baseline (***n*** = 15)	After 24 h (***n*** = 15)	After 48 h (***n*** = 15)
Age, years	64 (54–69)	–	–
Male gender	11 (73.3)	–	–
Angiotensin II as first-line agent	10 (66.7)	–	–
Angiotensin II dose, ng/kg/min	20.0 (5.0–20.0)	20.0 (8.4–20.8)	20.0 (8.1–20.8)
**Support and drugs**			
High dose catecholamine (> 0.25 μg/kg/min)	1 (6.7)	–	–
Receiving catecholamine > 12 h	2 (13.3)	–	–
Prone positioning	5 (41.7)	11 (78.6)	11 (78.6)
Use of tocilizumab	5 (35.7)	–	–
Norepinephrine dose, μg/kg/min	0.10 (0.10–0.20)	0.02 (0.00–0.09)	0.01 (0.00–0.14)
Hours using before	8.5 (1.8–15.8)	–	–
**Vital signs at start**			
Systolic arterial pressure, mmHg	110 (95–115)	110 (105–129)	120 (115–120)
Diastolic arterial pressure, mmHg	60 (52–64)	60 (56–64)	70 (59–70)
Mean arterial pressure, mmHg	71 (65–79)	77 (76–80)	85 (80–87)
Heart rate, bpm	82 (70–92)	72 (68–83)	71 (66–76)
Atrial fibrillation	1 (7.1)	–	–
Cumulative urine output, mL	237.5 (71.2–365.0)	620.0 (385.0–750.0)	727.0 (470.0–1050.0)
Oliguria	3 (30.0)	–	–
**Ventilatory support**			
FiO_2_	0.70 (0.61–0.70)	0.50 (0.40–0.60)	0.40 (0.36–0.54)
PEEP, cmH_2_O	14 (12–15)	12 (10–12)	11 (10–14)
SpO_2_, %	97 (94–99)	98 (96–98)	97 (91–98)
PaO_2_/FiO_2_	121.4 (98.1–218.1)	195.2 (148.3–245.0)	200.0 (168.0–248.5)
SpO_2_/FiO_2_	140.7 (132.5–150.6)	191.5 (118.4–258.0)	193.8 (142.2–235.9)
**Laboratory tests at start**			
Lactate, mmol/L	1.49 (1.36–1.56)	1.72 (1.58–2.00)	1.83 (1.53–2.15)
Creatinine, mg/dL	1.00 (0.85–1.68)	1.69 (1.16–2.38)	1.69 (1.06–2.43)
C-reactive protein, mg/dL	232.3 (165.4–269.2)	202.0 (148.4–231.1)	115.0 (95.0–190.4)
White blood cell count, × 1000 cells/mm^3^	11.9 (7.7–13.2)	10.1 (6.2–12.4)	9.2 (7.2–14.2)
Lymphocyte count, × 1000 cells/mm^3^	5.30 (3.05–16.222)	7.90 (3.70–12.85)	8.30 (5.20–13.50)

Data are median (quartile 25% to quartile 75%) or *N* (%)

*PEEP* positive end-expiratory pressure

**Fig. 1:**
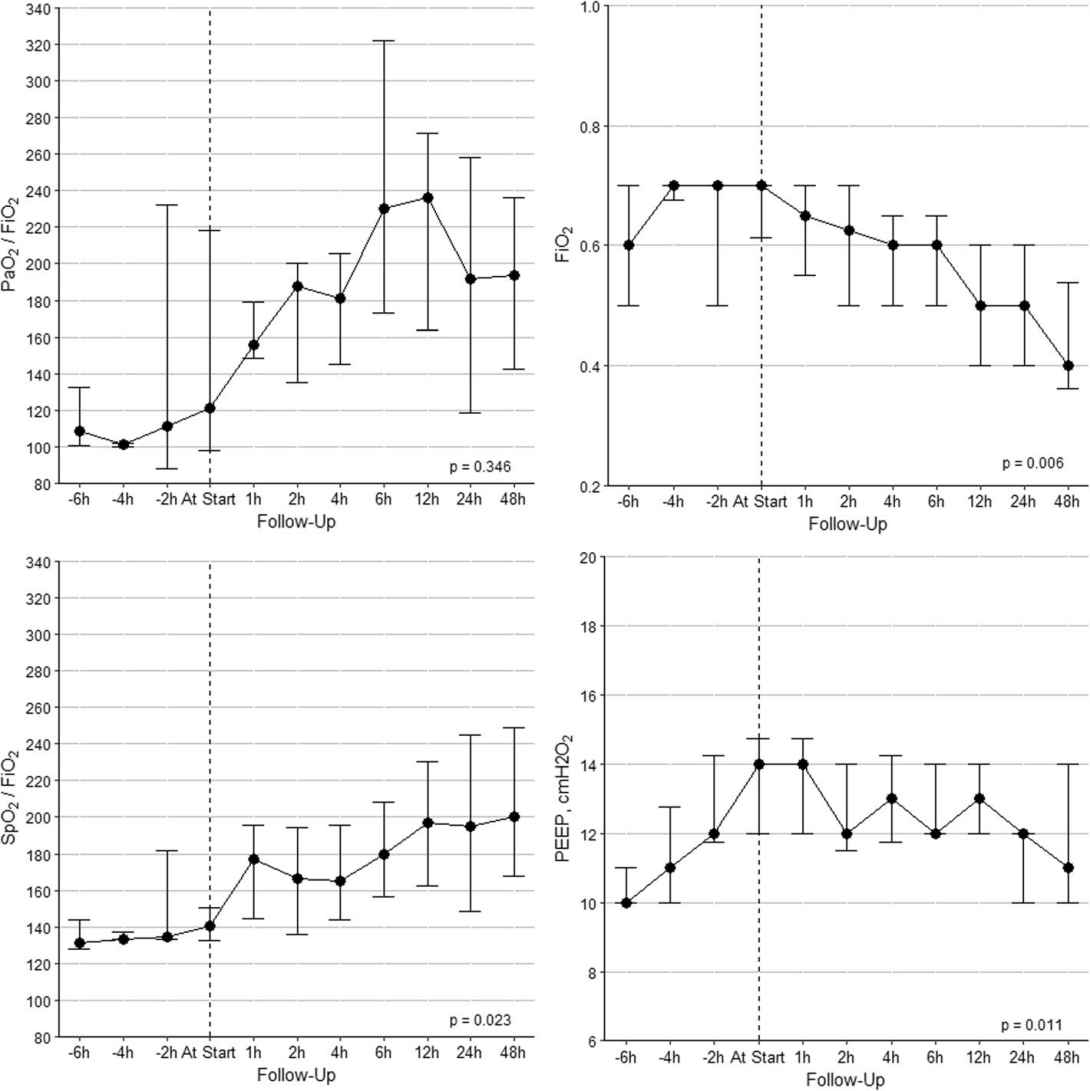
Changes in oxygenation parameters in the first 48 h of angiotensin II infusion. Data are median and quartile 25% to quartile 75%. The changes in the parameters over time were assessed with a mixed–effect quantile model based on the asymmetric Laplace distribution (*τ* = 0.50, a median regression), taking into account repeated measurements and considering the time of measurements (as a continuous variable) as fixed effect. The *p* value in the graphs represents the changes over this time. In all models, only values at and after the start of the infusion drug were taken into account, and the values before the start were used only for graphic purpose. All results were confirmed after bootstrapping with 10,000 replications. All analyses were conducted in R (R Foundation), version 3.6.3

In ventilated patients with COVID-19-associated vasodilatory shock, we assessed the initial physiological changes associated with ANGII infusion as primary or rescue vasopressor. Overall, the administration of ANGII was associated with achievement and maintenance of target MAP, an increase on SpO_2_/FiO_2_ ratio, and a decrease in FiO_2_. These oxygenation improvements were significant.

This represents the first experience with ANGII in COVID-19-associated vasodilatory shock and with ANGII as primary vasopressor in humans. The findings are consistent with those of a previous trial and subsequent subgroup [2] and ANG I/II ratio-related analyses [3]. They suggest the absence of early physiologically harm and improved oxygenation with ANG II.

The key limitations of this study are obvious. It is single-center, small, observational in nature; lacks a control population; and is open-label. However, in this pandemic setting, the ethics of ensuring compassionate drug use to all patients were considered a priority. Moreover, before considering controlled trials, evidence of some physiological safety was considered important. Finally, under the extraordinary pressures of the most dramatic health disaster in Italy’s history in a century, this study was the best possible under the circumstances.

In conclusion, we provide the first observational cohort study of ANGII infusion in ventilated patients with COVID-19-associated vasodilatory shock. Our findings provide preliminary evidence to assist clinicians in their choice of vasopressors and justify and help design future controlled studies.

## Data Availability

Full de-identified dataset and codes of the analyses are available upon request to the corresponding authors.
